# Dual Kidney Transplantation Offers Prolonged Graft Survival

**DOI:** 10.1111/ctr.70481

**Published:** 2026-02-11

**Authors:** Ekaterina Fedorova, Sofia Nehring Firmino, David Foley, Jacqueline Garonzik‐Wang, Dixon Kaufman, Jon Odorico, David Aufhauser, Nikole A. Neidlinger, Carrie Thiessen, Jennifer Philip, Kelly M. Collins, Josh Mezrich, David Al‐Adra, Didier Mandelbrot, Brad C. Astor, Sandesh Parajuli

**Affiliations:** ^1^ Division of Transplant Surgery Department of Surgery University of Wisconsin School of Medicine and Public Health Madison Wisconsin USA; ^2^ University of Wisconsin‐Madison School of Medicine and Public Health Madison Wisconsin USA; ^3^ Division of Nephrology Department of Medicine University of Wisconsin School of Medicine and Public Health Madison Wisconsin USA; ^4^ Department of Population Health Sciences University of Wisconsin School of Medicine and Public Health Madison Wisconsin USA

**Keywords:** donors and donation, graft survival, rejection: acute

## Abstract

**Introduction:**

Dual kidney transplantation (DKT), an uncommonly performed procedure, provides a unique opportunity to transplant nonstandard kidneys that might otherwise not be utilized. We compared perioperative and five‐year posttransplant outcomes between DKT, and single kidney transplants (SKT) performed at our institution.

**Methods:**

We analyzed all adult deceased donor kidney‐alone transplant recipients at our center between 2001 and 2020. Recipients of pediatric *en bloc* kidney transplants were excluded. Perioperative outcomes of interest included delayed graft function (DGF), posttransplant length of stay (LOS), rehospitalization, and reoperation. Five‐year outcomes included biopsy‐proven acute rejection (AR), death‐censored graft failure (DCGF), uncensored graft failure (UCGF), and death with functioning graft (DWFG).

**Results:**

A total of 100 DKT and 3125 SKT recipients were included. DKT recipients were older (*p* < 0.001), more often male (68%), and more often underwent early steroid withdrawal (*p* = 0.04). In comparison to SKT, after adjustment for multiple variables, DKT was not independently associated with DGF (aOR: 1.25; 95% CI 0.76–2.08); prolonged LOS (linear coefficient 0.42; −0.9–1.7); reoperation (aOR: 0.73; 95% CI: 0.21–2.51) or rehospitalization (aOR 0.98; 95% CI: 0.55–1.74). However, within five years, DKT had a lower adjusted incidence rate ratio (aIRR) for AR (aIRR: 0.28; CI 0.12–0.64); DCGF (aIRR: 0.30; 95% CI 0.13–0.68), and UCGF (aIRR: 0.53; 95% CI: 0.33–0.86), without statistically significant differences in DWFG (aIRR: 0.83; 95% CI: 0.46–1.53).

**Conclusion:**

In selected recipients, DKT offered superior medium‐term outcomes compared to SKT without compromising perioperative outcomes. DKT can mitigate concerns associated with medically complex donor kidneys, increase organ utilization, and increase access to transplantation.

## 1 Introduction

Kidney transplantation offers improved outcomes when compared to other renal replacement therapies, including increased quality of life, decreased number of cardiovascular events, and decreased mortality [1, 2, 3]. As such, it has become the treatment of choice for patients with end‐stage renal disease (ESRD). However, there remains a significant discrepancy between the number of potential transplant candidates and the annual transplants performed. In the United States, 28 142 kidney transplants were performed in 2023, while 46 661 new patients were listed for transplantation. [4]. As organ demand grows, the use of kidneys from medically complex donors has been pursued to expand the donor pool. [5, 6, 7].

Despite increased utilization of nonstandard kidneys, the proportion of donor kidney non‐utilization grows [4, 7, 8]. Dual kidney transplantation (DKT) is one surgical approach that helps mitigate concerns about donor medical complexity and nephron mass and possibly decreases non‐utilization. DKT was first performed in the United States in 1993 by Dr. Lloyd E. Ratner [9]. DKT was then first described in the literature in 1996 by Dr. Lynt B. Johnson [10, 11, 12]. DKT involves transplanting both kidneys from a single adult deceased donor into one recipient [10, 13]. The rationale for transplantation of both organs into one recipient is that two nonstandard kidneys could achieve the equivalent functioning nephron mass to that of a single kidney from a less medically complex donor [13, 14, 15, 16, 17].

**FIGURE 1:**
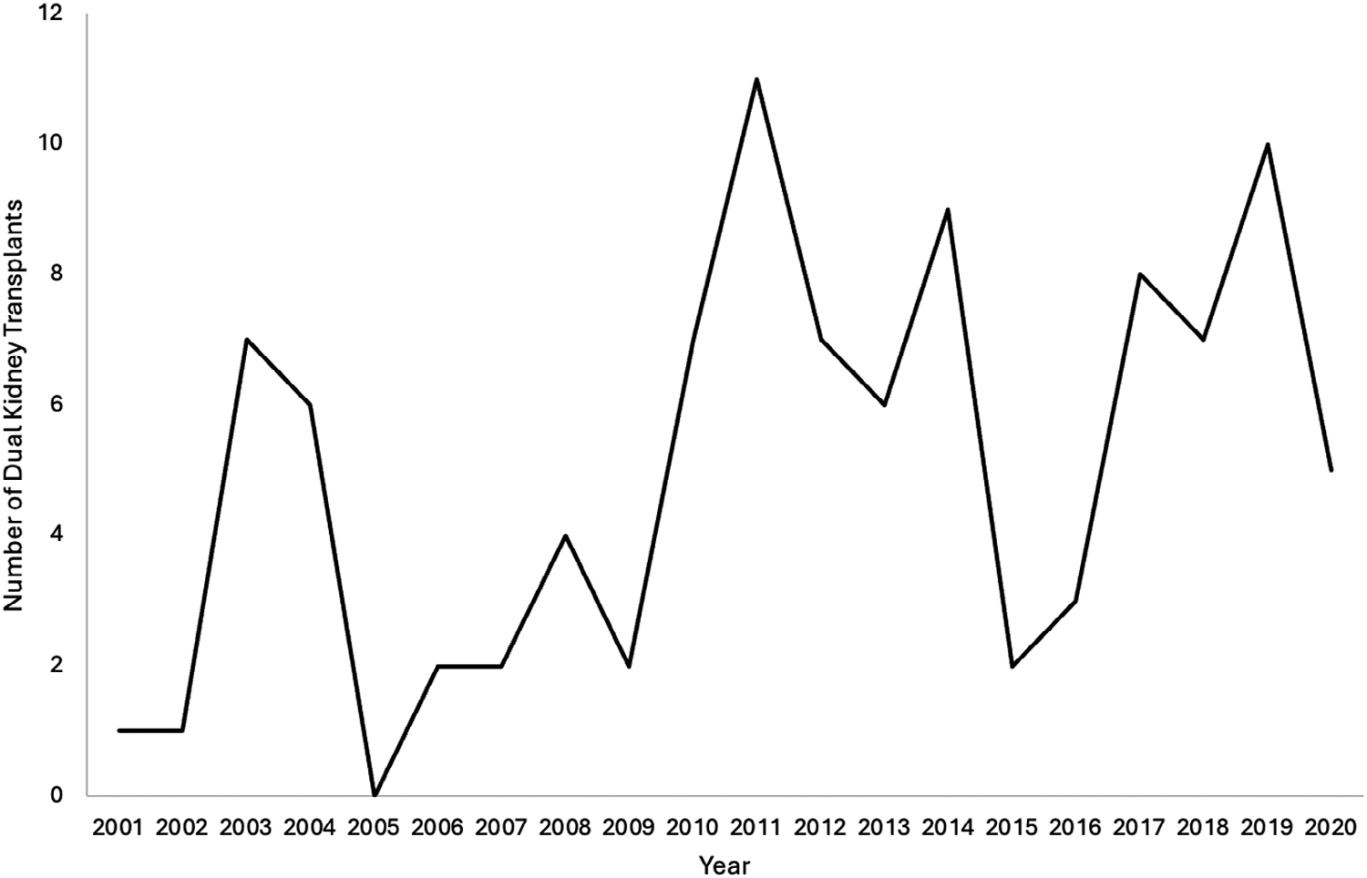
Number of DKTs performed by year at the University of Wisconsin‐Madison.

In the United States, DKT is an uncommonly performed approach to renal transplantation, comprising only 4% of kidney transplants from 2000 to 2005, with only 46 transplant centers performing DKTs between September of 2019 and August of 2021 [18, 19]. In a recent study utilizing an Organ Procurement and Transplantation Network dataset, Kaufman et al. reported 1,015 DKT recipients between 12/2014 and 03/2024 in the United States, averaging approximately 110 per year [20]. At our center, between 2001 and 2020, zero to eleven DKTs were performed per year, as shown in Figure 1. As the demand for kidney transplantation increases, DKT offers a unique opportunity to utilize high kidney donor profile index (KDPI) kidneys, especially if post‐DKT outcomes were suitable. We sought to explore and describe perioperative and long‐term post‐DKT outcomes and compare them to single‐kidney transplantation (SKT) outcomes at our institution over a 2‐decade time period.

## 2 Methods

### 2.1 Population Selection and Study Design

We studied all adult kidney transplant recipients of deceased donor kidneys between 2001 and 2020 at our center. We excluded recipients of a pediatric *en bloc* kidney transplant and recipients who had a prior non‐kidney transplant. Recipients were divided into two cohorts based on whether they received a single or dual kidney transplant.

Perioperative outcomes of interest included delayed graft function (DGF), hospital length of stay (LOS) after the index transplant, rehospitalization within 30 days of discharge, and reoperation due to transplant surgical complications within 30 days. Five‐year outcomes of interest included biopsy‐proven acute rejection (AR), death‐censored graft failure (DCGF), uncensored graft failure (UCGF), and death with functioning graft (DWFG). DGF was defined as the need for dialysis within one week of transplant. Additionally, we compared kidney function between DKT and SKT recipients by assessing serum creatinine and estimated glomerular filtration rate utilizing the 2021 race‐free CKD‐EPI Creatinine Equation at 3 and 6 months posttransplant, as well as at 1, 3, and 5 years.

This study was approved by the University of Wisconsin School of Medicine and Public Health Institutional Review Board (IRB protocol number: 2014‐1072). This study followed the Declaration of Helsinki. The clinical and research activities being reported were consistent with the Principles of the Declaration of Istanbul as outlined in ‘The Declaration of Istanbul on Organ Trafficking and Transplant Tourism’. Due to the nature of this study, informed consent explicit to this research was not obtained from patients.

### 2.2 Adult Dual Kidney Allocation

Allocation of organs for DKT varied during our study period. From 2001 to 2013, our institution functioned as a single‐center organ procurement organization, and allocation of dual kidneys from our donation service area (DSA) was determined by center‐specific decision‐making. Changes in kidney allocation policies between 2014 and 2019 resulted in less availability of organs appropriate for DKT due to broader allocation outside our DSA. As of 2019, our center follows the Organ Procurement and Transplantation Network's dual kidney allocation policy [21].

Even though allocation policies varied during the study period, the decision to transplant donor kidneys as duals was determined by kidney biopsy and scoring described by Remuzzi et al. [22, 23]. Additionally, throughout the study period, our center did not have a formal programmatic cold ischemia time (CIT) cutoff for DKTs, and DKT allocation was based on the accepting surgeon's discretion. Patients were designated as candidates for dual kidney implantation after being reviewed at our multidisciplinary kidney selection committee meeting. Recipient candidates were determined based on urgency of need for transplant, degree of iliac atherosclerotic disease, visceral adiposity, adequate retroperitoneal space, particularly in patients with polycystic kidney disease, and absence of comorbidities, such as recipient age, primary transplant status, and body mass index (BMI) less than 35 kg/m^2^, that would significantly increase the risk of a longer and more complex surgery.

### 2.3 Immunosuppression and Clinic Follow‐up

At our center, kidney transplant recipients underwent immunosuppression induction with antithymocyte globulin, alemtuzumab, or basiliximab, followed by immunosuppression with tacrolimus, and mycophenolic acid, with or without prednisone, as previously described [24]. Prophylaxis for pneumocystis pneumonia was with trimethoprim‐sulfamethoxazole for 12 months posttransplant. Cytomegalovirus prophylaxis with valganciclovir was initiated within 72 h of transplant in previously described high‐risk populations [24, 25]. Doses of trimethoprim‐sulfamethoxazole varied based on renal function and ranged between 160 and 800 mg daily to three times a week [26].

After patients were discharged from their initial transplant admission, patients received follow‐up at either the University Hospital or at one of multiple outreach regional clinics. Patients were seen at 3 and 6 weeks; 6, 9, 12, 18, and 24 months postoperatively; and annually thereafter. Patients underwent routine laboratory testing. Additionally, our transplant clinic managed all major health events as previously described [24].

### 2.4 Surgical Technique

At our center, our standard approach to SKTs was to place the donor kidney retroperitoneally into the recipient's iliac fossa with occasional variations as needed based on recipient anatomy. In those cases, the donor kidney was placed intraperitoneally. The allograft's renal artery and vein were anastomosed to the recipient's common or external iliac vessels, regardless of whether the allograft was placed intraperitoneally or retroperitoneally. The donor ureter was then anastomosed to the recipient's urinary bladder via a ureteroneocystostomy.

During the study period, DKT recipients underwent either a unilateral (71%) or bilateral (29%) transplant. In a unilateral transplant, both donor kidneys were positioned into one of the recipient's iliac fossae. This approach was most often extraperitoneal (87.3%) and through a right lower quadrant transplant, or Gibson, incision (90.1%). The proximal, or cephalad, kidney was typically implanted first with anastomosis to the common iliac vessels or proximal external iliac vessels, followed by implantation of the distal, or caudal, kidney to the distal external iliac vessels.

In the case of a bilateral transplant, the kidney allografts were positioned into separate iliac fossae via a midline incision with an intraperitoneal approach (86%) or bilateral transplantation incisions via an extraperitoneal approach (14%). Each graft's vasculature was anastomosed to the iliac vasculature of its corresponding side. Regardless of approach, ureters were implanted separately or together into the recipient's urinary bladder. All organs, both for SKTs and DKTs, were preserved with UW solution.

### 2.5 Statistical Analysis

Baseline characteristics were compared using chi‐square or *t* tests, as appropriate. The baseline characteristics including donor factors, immunologic factors, and recipient factors listed in Table 1 were included in multivariable analysis for adjustment. Unadjusted Kaplan‐Meier survival analysis for some outcomes of interests was created comparing SKT and DKT. Due to baseline differences between the study groups, an analysis excluding both DKT and SKT recipients who had a history of previous kidney transplantation, cPRA greater than 50%, and BMI greater than 40 kg/m^2^ were excluded and analysis were repeated for the same outcomes of interest with adjustment for baseline characteristics listed in Table 1. A *p*‐value of ≤0.05 was considered statistically significant. All analyses were conducted using Stata software (Version SE 15; StataCorp LLC, College Station, TX).

**TABLE 1:** Baseline characteristics of transplant recipients and donors.

	Variables	Dual kidney (*n* = 100)	Single deceased donor (*n* = 3125)	*p* value
Donor factors	Mean age (years)	60.8 (10.2)	42.2 (15.9)	<0.001
	Male (%)	54.0	38.3	0.002
	Non‐white (%)	6.0	8.5	0.38
	Mean BMI (Kg/m^2^)	28.5 (6.9)	28.5 (7.6)	0.97
	Cause of death: Cardiovascular (%)	62.0	30.9	<0.001
	Terminal serum creatinine (mg/dl)	1.08 (0.49)	0.99 (0.78)	0.29
	Mean KDPI (%)	78.8 (16.6)	42.2 (25.7)	<0.001
	DCD (%)	22.0	28.9	0.14
	Mean kidney cold ischemia time (h)	14.6 (5.9)	16.7 (6.2)	0.001
Immunologic factors	cPRA > 20% (%)	13.0	27.9	0.002
	Mean HLA mismatch (of 6)	4.3 (1.4)	3.9 (1.6)	0.02
	Previous transplant (%)	2.0	20.4	<0.001
Recipients factors	Mean age (years)	56.9 (9.8)	52.3 (12.7)	<0.001
	Female (%)	32.0	38.9	0.16
	Non‐white (%)	20.0	27.4	0.10
	Mean BMI (Kg/m^2^)	28.2 (5.1)	28.0 (5.3)	0.66
	Cause of ESRD	—	—	0.57
	Diabetes	32.0	26.9	
	Hypertension	17.0	14.1	
	Glomerulonephritis	11.0	11.5	
	Polycystic kidney disease	24.0	24.7	
	Other	16.0	22.8	
	Induction immunosuppression (%)	—	—	0.57
	Alemtuzumab	23.0	19.2	
	Antithymocyte globulin	29.0	28.2	
	Basiliximab	48.0	52.4	
	Early steroid withdrawal (%)	11.0	6.0	0.04
	Preemptive transplant	10.0	12.7	0.42

## 3 Results

One hundred patients underwent an adult DKT, and 3125 patients had an adult SKT (Table 1). The mean (± standard deviation) age for DKT recipients was 56.9 ± 9.8 years, while the mean (± standard deviation) age for the SKT recipient was 52.3 ± 12.7 years (*p* < 0.001). Sixty‐eight percent of DKT recipients were male. The primary cause for ESRD for both groups was diabetes mellitus. Induction with basiliximab was used in 48% of DKT cases, and 52% of SKT cases. Early steroid withdrawal protocol was more common in the DKT group (11%) when compared to the SKT group (6%), *p* = 0.04. Only 2% of DKT patients had previously received a prior transplantation, while 20% of SKT recipients had a prior kidney transplant (*p* < 0.001). Recipients with a cPRA >20% were higher in the SKT group (27.9%) when compared to the DKT group (13%), *p* = 0.002.

In terms of donor characteristics, donors were older (61 years old vs. 42 years old, *p* < 0.001) and more often males in the DKT group when compared to SKT (54.0% vs. 38.2%, *p* = 0.002). Additionally, mean KDPI was higher in the DKT group (78.8%) compared to the SKT group (42.2%). Cause of death due to cardiovascular collapse was more prevalent in the donors in the DKT group (62% vs. 30.9%, *p* < 0.001). There were more donation after circulatory death (DCD) donors in the SKT group, although not statistically significant (28.9% vs. 22.0%, *p* = 0.14). CIT was shorter in the DKT group at 14.6 h compared to 16.7 h in the SKT group (*p* = 0.001). Of note, 61 DKT donors were local, and the remaining 39 were imported kidneys from outside Organ Procurement Organizations.

### 3.1 Perioperative Complications

DKT recipients were not associated with an increased risk of perioperative complications, including DGF, reoperation, rehospitalization, and LOS when compared to SKT recipients (Table 2). While DKT recipients had a higher risk of DGF [odds ratio (OR): 1.54, 95% CI: 1.02–2.32, *p* = 0.04], after adjustment for donor and recipient variables, the risk of DGF was comparable between the two groups [adjusted odds ratio (aOR): 1.25; 95% CI: 0.76–2.08, *p* = 0.38]. Additionally, DKT was not associated with increased LOS both before (linear coefficient 1.2, −0.1 to 2.5, *p* = 0.67) and after adjustment for baseline characteristics (linear coefficient 0.42, −0.9 to 1.7, *p* = 0.52). DKT was not associated with increased rehospitalizations (aOR: 0.98; 95% CI: 0.55–1.74, *p* = 0.94). DKT recipients were not associated with an increased risk of reoperation both before (OR: 1.75, 95% CI: 0.74–4.08, *p* = 0.20) and after adjustment for baseline characteristics (aOR: 0.73, 95% CI: 0.21–2.51, *p* = 0.62). Most common indications for reoperation included postoperative bleeding and infection. During the study period, one DKT recipient experienced early graft failure on posttransplant day 2 due to dual graft thrombosis. There were no cases of single graft thrombosis in DKT patients.

**TABLE 2:** Events within 5 years of transplantation: DGF, reoperations, rehospitalizations, LOS.

		Dual	Single	*p*
DGF	# / *N*	39/100 (39.0%)	916/3125 (29.3%)	—
	OR	1.54 (1.02, 2.32)	Reference	0.04
	aOR^a^	1.25 (0.76, 2.08)	Reference	0.38
Reoperations	# / *N*	6/100 (6.0%)	110/3125 (3.5%)	—
	OR	1.75 (0.75, 4.08)	Reference	0.20
	aOR^a^	0.73 (0.21, 2.51)	Reference	0.62
Rehospitalizations	# / *N*	18/100 (18.0%)	515/3125 (16.5%)	—
	OR	1.11 (0.66, 1.87)	Reference	0.67
	aOR^a^	0.98 (0.55, 1.74)	Reference	0.94
LOS	Mean number of days (SD)	9.3 (7.4)	8.1 (6.5)	0.12
	Linear coefficient	1.2 (−0.1, 2.5)	—	0.67
	Linear coefficient (adjusted^a^)	0.42 (−0.9, 1.7)	—	0.52

^a^ Adjusted for all variables in Table 1.

To address heterogeneity in SKT and DKT recipients, we repeated the data analysis after excluding patients who were re‐transplants (*N* = 637), had a cPRA greater than 50% (*N* = 263), and had a BMI greater than 40 kg/m^2^ (*N* = 24). A total of 89 DKT recipients and 2201 SKT recipients were included (Table S1). Similarly to our previous data analysis, DKT recipients had a higher risk of DGF (OR: 1.54, 95% CI: 1.00–2.38, *p* = 0.05), but after adjustment for donor and recipient variables, the risk of DGF was comparable between the two groups (aOR: 0.39; 95% CI: 0.74–2.20, *p* = 0.39). The risk of reoperations (OR: 1.66; 95% CI: 0.66–4.22, *p* = 0.28) and rehospitalization (OR: 0.97; 95% CI: 0.74–2.17, *p* = 0.39) was comparable between the two groups in unadjusted analysis. After adjustment for donor and recipient variables, the risk of reoperations (aOR: 0.73; 95% CI: 0.16–3.26, *p* = 0.68) and rehospitalizations (aOR: 0.97; 95% CI: 0.52–1.78, *p* = 0.91) remained comparable between DKT and SKT recipients. Unlike the previous analysis, on repeat analysis DKT was associated with an increased LOS before adjustment for baseline characteristics (linear coefficient: 1.42, −0.02 to 1.78, *p* = 0.05). However, following adjustment for baseline characteristics, LOS was comparable between the two groups (linear coefficient: 0.54, −0.95 to 2.03, *p* = 0.48).

### 3.2 Five‐Year Complications

Within 5 years, DKT recipients were less likely to experience AR and DCGF, reflecting improved graft survival (Table 3). At 5 years, the UCGF incidence was 28% in DKT recipients, corresponding to an unadjusted graft survival (UAGS) of 72% compared to a UCGF incidence of 26% and UAGS of 74% in SKT recipients. The rate of UCGF was higher in the DKT group, with 7.26 per 100 person‐years compared to the 6.52 per 100 person‐years in the SKT group. The incidence of DCGF was similar between DKT and SKT recipients, 12% and 12.7%, respectively. The rate of DCGF was also similar between the DKT and SKT groups, at 3.11 and 3.21 per 100 person‐years, respectively. The rate of DWFG was slightly higher in the DKT group at 4.15 per 100 person‐years compared to that of the SKT group of 3.32 per 100 person‐years. Additionally, 9% of DKT recipients developed AR compared to 21.1% of SKT recipients, with an incidence rate per 100 person‐years of 2.43 and 6.28, respectively.

**TABLE 3:** Events within 5 years of transplantation: AR, DCGF, UCGF, DWFG.

		Dual	Single	*p*
UCGF	# / *N*	28/100 28%	812/3125 (26%)	—
	Incidence rate (/100 person‐years)	7.26	6.52	—
	IRR	1.11 (0.76, 1.62)	Reference	0.59
	aIRR^a^	0.53 (0.33, 0.86)	Reference	0.01
DCGF	# / *N*	12/100 (12%)	399/3125 (12.8%)	—
	Incidence rate (/100 person‐years)	3.11	3.21	—
	IRR	0.96 (0.54, 1.71)	Reference	0.90
	aIRR^a^	0.30 (0.13, 0.68)	Reference	0.004
DWFG	# / *N*	16/100 (16%)	413/3125 (13.2%)	—
	Incidence rate (/100 person‐years)	4.15	3.32	—
	IRR	1.25 (0.76, 2.06)	Reference	0.38
	aIRR^a^	0.83 (0.46, 1.53)	Reference	0.56
AR	# / *N*	9/100 (9%)	661/3125 (21.1%)	—
	Incidence rate (/100 person‐years)	2.43	6.28	—
	IRR	0.40 (0.21, 0.77)	Reference	0.006
	aIRR^a^	0.28 (0.12, 0.64)	Reference	0.002

^a^ Adjusted for all variables in Table 1.

**FIGURE 2:**
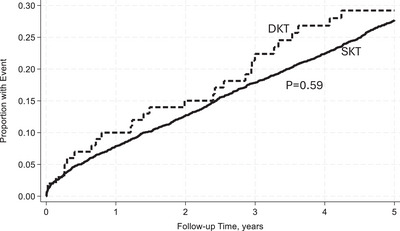
Cumulative incidence of UCGF between DKT and SKT within 5 years posttransplant unadjusted for baseline characteristics.

**FIGURE 3:**
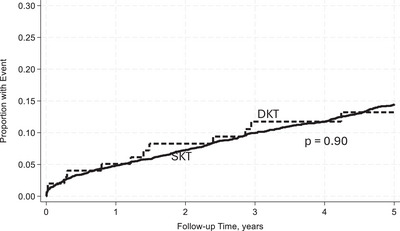
Cumulative incidence of DCGF between DKT and SKT within 5 years posttransplant unadjusted for baseline characteristics.

**FIGURE 4:**
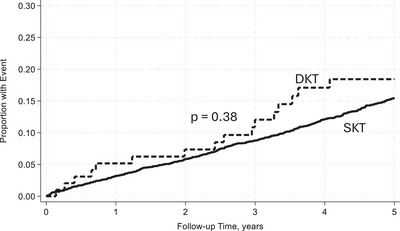
Cumulative incidence of DWFG between DKT and SKT within 5 years posttransplant unadjusted for baseline characteristics.

**FIGURE 5:**
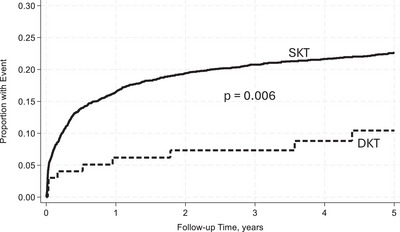
Cumulative incidence of AR between DKT and SKT within 5 years posttransplant unadjusted for baseline characteristics.

With reference to SKT, in unadjusted analysis DKT recipients had a similar rate of UCGF [incidence rate ratio (IRR): 1.11, 95% CI: 0.76–1.62, *p* = 0.59] (Figure 2). However, when adjusted for donor and recipient characteristics, DKT was associated with a lower rate of UCGF [adjusted incidence rate ratio (aIRR): 0.53, 95% CI: 0.33–0.86, *p* = 0.01)]. Additionally, DKT was not associated with an increased rate of DCGF in unadjusted analysis (IRR: 0.96, 95% CI: 0.54–1.71, *p* = 0.90) (Figure 3). After adjusting for baseline characteristics, DKT was associated with a lower rate of DCGF (aIRR: 0.30, 95% CI: 0.13–0.68, *p* = 0.004). DKT was not associated with an increased rate of DWFG both before and after adjustment for baseline characteristics (aIRR: 0.83, 95% CI: 0.46–1.53, *p* = 0.56) (Figure 4). With reference to SKT, DKT recipients experienced a significantly lower rate of AR (IRR): 0.40; 96% CI: 0.21–0.77; *p* = 0.006 (Figure 5). The lower rate of AR seen in DKT remained after adjustment for donor and recipient factors (aIRR: 0.28; 95% CI: 0.12–0.64, *p* = 0.002).

On subgroup analysis excluding patients with increased immunologic and operative risk and adjusting for baseline characteristics listed on Table 1, the same relationships were observed between DKT and SKT recipients (Table S2). DKT recipients were less likely to experience UCGF, DCGF, and AR. With reference to SKT, DKT recipients experienced a similar rate of UCGF before adjustment for baseline characteristics (IRR: 1.28; 95% CI: 0.87–1.86, *p* = 0.21). However, after adjustment, DKT recipients were less likely to experience UCGF (aIRR: 0.57, 95% CI: 0.35–0.93, *p* = 0.03). DKT recipients experienced a comparable rate of DCGF with reference to SKT recipients (IRR: 1.11; 95% CI: 0.62–1.98, *p* = 0.72). After adjusted analysis, DKT was associated with a lower rate of DCGF (aIRR: 0.31, 95% CI: 0.13–0.71, *p* = 0.006). With reference to SKT, DKT was not associated with a lower rate of DWFG in both unadjusted (IRR: 1.43; 95% CI: 0.87–2.37, *p* = 0.16) and adjusted (aIRR: 0.93, 95% CI: 0.50–1.71, *p* = 0.80) analysis. However, DKT continued to be associated with a lower rate of AR both in unadjusted (IRR: 0.43; 95% CI: 0.22–0.87, *p* = 0.02) and adjusted analysis (aIRR: 0.2, 95% CI: 00.10–0.62, *p* = 0.003).

### 3.3 Kidney Function

Recipient creatinine and glomerular filtration rates (GFR) were similar between DKT and SKT recipients. At 6 months follow‐up, DKT recipient creatinine was lower than that of SKT recipients (1.47 mg/dL vs. 1.56 mg/dL, *p* = 0.26). This relationship was observed at all follow‐up times of 1 year, 3 years, and 5 years (Table 4). However, the difference in creatinine values was not statistically significant at any of the follow‐up points. GFRs between the two groups were also not statistically significant at any of the follow‐up points (Table 5).

**TABLE 4:** Creatinine (SD) at follow‐up times.

	Dual	Single	*p* Value
3 months^a^	*N* = 98 1.55 (0.75) mg/dL	*N* = 2985 1.56 (0.60) mg/dL	0.48
6 months^a^	*N* = 90 1.47 (0.54) mg/dL	*N* = 2887 1.56 (0.78) mg/dL	0.26
1 year^b^	*N* = 87 1.45 (0.52) mg/dL	*N* = 2771 1.58 (1.14) mg/dL	0.32
3 years^b^	*N* = 67 1.43 (0.77) mg/dL	*N* = 2175 1.53 (0.93) mg/dL	0.37
5 years^b^	*N* = 50 1.39 (0.53) mg/dL	*N* = 1594 1.55 (1.16) mg/dL	0.33

^a^ ± 1 month.

^b^ ± 2 months.

**TABLE 5:** Estimated GFR (SD) at follow‐up times.

	Dual	Single	*p* Value
3 months^a^	*N* = 96 58.6 (22.7) mL/min	*N* = 2983 56.1 (20.8) mL/min	0.24
6 months^a^	*N* = 91 57.2 (20.3) mL/min	*N* = 2887 55.7 (20.3) mL/min	0.48
1 year^b^	*N* = 87 59.5 (22.9) mL/min	*N* = 2771 56.6 (20.9) mL/min	0.21
3 years^b^	*N* = 67 62.2 (22.4) mL/min	*N* = 2175 59.2 (23.0) mL/min	0.30
5 years^b^	*N* = 50 62.9 (23.7) mL/min	*N* = 1594 59.8 (23.8) mL/min	0.37

^a^ ± 1 month.

^b^ ± 2 months.

## 4 Discussion

In this large cohort of 3225 deceased donor kidney transplant recipients, 100 (3.3%) received a DKT. Compared to SKT, DKT recipients had similar early postoperative complications, such as DGF, LOS, reoperation rate, and rehospitalization. Additionally, DKT was associated with a decreased risk of AR, DCGF, and UCGF. DKT was also associated with similar rates of DWFG when compared to SKT. In all, these results suggest that DKT offers prolonged graft survival when compared to SKT.

Previously, DKT has been shown to have similar outcomes, including DGF rates, graft survival, and rejection rates when compared to SKT [12, 18, 27, 28, 29, 30, 31, 32, 33]. DKT has also been reported to have similar rates of postoperative complications to that of SKT recipients [23, 32, 34, 35]. In a study by Das et al., the authors reported increased operative times in the DKT group but no increased risk of postoperative complications or prolonged LOS [32]. Khalid et al. also found similar kidney function and graft survival between DKT and SKT recipients [33]. Contrary to our findings, however, they reported that their DKT recipients were more likely to develop postoperative surgical complications requiring reoperation and longer lengths of hospital stay. The authors hypothesized the higher rate of surgical complications may be secondary to their small sample size of 34 DKTs during the study period, leading to an inability to generalize these findings [33].

In a larger study with a cohort of 100 DKTs, Ekser et al. reported no difference in the rate of surgical complications and hospital stay between DKT and SKT recipients, despite significantly longer operative times (260 vs. 157 min) [34]. In our study, DKT recipients were not associated with a risk of reoperation, increased LOS, or increased risk of rehospitalization compared to SKT recipients. Our findings, based on a larger cohort of 100 DKTs but with similar donor and recipient characteristics, operative, and immunosuppression protocols to that of Das et al., support the idea that a sufficiently larger sample size is needed to observe similar rates of surgical complications between the two groups [32].

DKT recipients in our experience had shorter CIT than SKT recipient, despite DKT allocation often occurring out of sequence. We attribute this difference to surgeon judgement in only considering dual kidney offers that occur relatively early in the process and allow ample time for the added complexity and operative time associated with DKT. In our study, despite shorter CIT, and higher mean HLA mismatching, older donor age, and higher KDPI values in the DKT group, the rate of DGF did not differ between groups. DGF is a well‐known complication of kidney transplantation, especially of high‐KDPI kidneys. It results from ischemia‐reperfusion injury and has been associated with worse outcomes in kidney transplant recipients, including infection, graft rejection, and lower graft function and patient survival [36, 37, 38]. DGF is often seen in nonstandard kidney recipients, as these organs often have risk factors for DGF, such as advanced donor age, cardiovascular comorbidities, longer warm or CIT, and DCD [39]. Based on these characteristics, it would be expected that DKT recipients would have higher rates of DGF, as DKT donors are often older and have higher KDPI values. However, in accordance with our results, previous studies reported no difference in the rate of DGF between DKT and SKT [12, 30, 31, 32, 33, 40, 41, 42, 43]. A minority of studies reported a significantly lower rate of DGF in DKT patients when compared to SKT [18, 35]. Gill et al. proposed that the lower DGF rate was due to increased nephron mass in two marginal kidneys compared to a single, high‐KDPI kidney [18]. The combined functional reserve from two kidneys likely offers greater resilience to ischemic injury and other stressors. This enhanced reserve enables better early graft function, making DKT recipients less susceptible to DGF. This further supports the hypothesis that increased nephron functional reserve plays a protective role in DKT outcomes.

Additionally, DKT was associated with a lower risk of graft failure, both DCGF and UCGF. Previously, graft survival in DKT had been reported to be comparable to that of SKT [10, 30, 31, 33, 44, 45, 46, 47]. Comparable graft and patient survival in DKT recipients were first described by Johnson et al. in 1996. The authors reported 100% graft and patient survival in DKT compared to 95% graft and patient survival in SKT from donors less than 50 years old within 6 months of transplantation [10]. In a large national registry‐based study in the United Kingdom, Ibrahim et al. demonstrated comparable 5‐year death‐censored graft survival between DKT and SKT recipients despite higher donor age and KDPI of DKTs [46]. Similar to our study, Lucarelli et al. found that at 5 years posttransplant, graft survival was significantly higher in DKTs (89%) compared to SKTs (76%) [45]. Savoye et al. also reported improved graft and patient survival in DKT recipients, despite being from significantly older donors [47]. The potential mechanism for increased graft survival seen in human studies can be illustrated by animal studies. In a rodent study by Wang et al., mice underwent either a DKT or SKT, and their kidneys were analyzed 1 month postimplantation. The authors reported increased injury, inflammation, and hypertrophy in the SKT group compared to the DKT group. They hypothesize that DKT creates a more physiologic hemodynamic response, lowering hyperfiltration stress relative to single kidney recipients [48]. Overall, both immune‐mediated injury and hyperfiltration‐mediated injury to SKT recipients may contribute to cumulative insults, ultimately resulting in a higher rate of graft failure.

Similarly to our study, DKT has previously been associated with lower or comparable rates of AR [23, 30, 31, 33, 35, 40]. As seen in our study, DKT donors are typically older; and therefore, it is possible that the decrease in AR seen is secondary to a decreased load of antigens per kidney secondary to a decreased number of immune cells with aging and aging‐related immune senescence [49, 50]. In our study, another finding that could explain this finding is the previous history of kidney transplantation in SKT recipients (20.4%) compared to DKT recipients (2%). SKTs also had a higher proportion of recipients with cPRA > 20% (27.9%) when compared to the DKT group (13%). A higher percentage of DKT recipients received induction immunosuppression with Alemtuzumab, a more depleting medication that could have aided with more profound immunosuppression, and therefore, the lower rate of AR was seen.

Unlike previous studies, our study is one of the largest single‐center studies analyzing the outcomes of DKT in the modern era. Our study provides a larger sample size of 100 DKTs when compared to other studies. Our study also had consistent patient follow‐up, providing more granular data than larger registries comprised of data from multiple centers. DKT is not a commonly performed procedure, and even in our high‐volume center the rate was extremely low. Moreover, DKT faces the challenge of the current organ allocation system. Organs considered for DKT have higher KDPI and/or biopsy findings suggestive of impaired renal function. These factors, in combination with extended CIT and increased operative times, may result in deferral of DKTs. However, our findings are consistent with previously published studies, both in the United States and internationally, which may further support the consideration of DKT in clinical practice and possibly updated, expedited organ allocation schemes for DKT.

This study has the expected limitations of a single‐center observational study, reflecting our specific population and clinical approach, which should be factored into the interpretation. Notable limitations in our study include the variability in organ allocation for DKTs and recipient selection for DKT throughout the study period, which we attempted to address through our statistical modeling and analysis. Likely due to a mismatch in sample sizes between the DKT and SKT groups, we noticed no statistically significant differences in the eGFR between the two groups, although higher eGFR in the DKT group at various time points could be clinically significant. Also, it was not possible to match all of the donor and recipient characteristics in the study, which may have introduced inherent bias. Additionally, we were unable to appropriately identify some of the immediate perioperative complications, such as urine leak, perinephric fluid collection, and ureteral stricture.

In conclusion, we demonstrate good short‐ and medium‐term outcomes with DKT, suggesting that, when appropriately used, it is a viable method to increase utilization of high‐DKPI kidneys. DKT should be considered prior to non‐utilization of donor kidneys to help mitigate organ shortage.

## Author Contributions

Ekaterina Fedorova: manuscript preparation, data collection, analysis, writing. Sofia Nehring Firmino: manuscript preparation, data collection, analysis, writing. David Foley: editing. Jacqueline Garonzik‐Wang: editing. Dixon Kaufman: editing. Jon Odorico: editing. David Aufhauser: editing. Nikole A. Neidlinger: editing. Carrie Thiessen: editing. Jennifer Philip: editing. Kelly M. Collins: editing. Josh Mezrich: editing. David Al‐Adra: editing. Didier Mandelbrot: editing. Brad C. Astor: analysis and editing. Sandesh Parajuli: original concept, design, writing, editing.

## Conflicts of Interest

The authors declare no conflicts of interest.

## Supporting information




**Supplemental Table 1**. Events within 5 years of transplantation: DGF, reoperations, rehospitalizations, LOS.

**Supplemental Table 2**: Events within 5 years of transplantation: AR, DCGF, UCGF, DWFG.

## Data Availability

The data that support the findings of this study are available from the corresponding author upon reasonable request.
