# Supplementary material for: Dual Kidney Transplantation Offers Prolonged Graft Survival
Source: Clin Transplant. 2026 Feb 11;40(2):e70481. doi: 10.1111/ctr.70481 (PMC12895095; doi:10.1111/ctr.70481)
Supplement: Supplementary file 1 — Supplemental Table 1. Events within 5 years of transplantation: DGF, reoperations, rehospitalizations, LOS. [file CTR-40-e70481-s001.docx]

**Supplemental Table 1. Events within 5 years of transplantation: Delayed Graft Function, Re-operations, Re-hospitalizations, Length of Stay**

*Adjusted for all variables in Table 1, after excluding recipients who were re-transplants, had BMI > 40 kg/m^2^, and had cPRA > 50%.

|  |  | Dual | Single | p |
| --- | --- | --- | --- | --- |
| Delayed Graft Function | # / N | 35/89 | 651/2201 | - |
|  | Odds Ratio | 1.54  (1.00, 2.38) | Reference | 0.05 |
|  | Odds Ratio (adjusted*) | 1.27  (0.74, 2.20) | Reference | 0.39 |
| Re-operations | # / N | 5/89 | 76/2201 | - |
|  | Odds Ratio | 1.66  (0.66, 4.22) | Reference | 0.28 |
|  | Odds Ratio (adjusted*) | 0.73  (0.16, 3.26) | Reference | 0.68 |
| Re-hospitalizations | # / N | 17/89 | 346/2201 | - |
|  | Odds Ratio | 1.27  (0.74, 2.17) | Reference | 0.39 |
|  | Odds Ratio (adjusted*) | 0.97  (0.52, 1.78) | Reference | 0.91 |
| Length of Stay | Mean (SD) | 9.53 (7.75) | 8.11 (6.73) | - |
|  | Linear coefficient | 1.42  (-0.02, 2.86) | - | 0.05 |
|  | Linear coefficient (adjusted*) | 0.54  (-0.95. 2.03) | - | 0.48 |
