# Supplementary material for: Dual Kidney Transplantation Offers Prolonged Graft Survival
Source: Clin Transplant. 2026 Feb 11;40(2):e70481. doi: 10.1111/ctr.70481 (PMC12895095; doi:10.1111/ctr.70481)
Supplement: Supplementary file 2 — Supplemental Table 2: Events within 5 years of transplantation: AR, DCGF, UCGF, DWFG. [file CTR-40-e70481-s002.docx]

**Supplemental Table 2: Events within 5 years of transplantation: Acute Rejection, Death-censored graft failure, Uncensored Graft Failure, Death with a Functioning Graft**

*Adjusted for all variables in Table 1, after excluding recipients who were re-transplants, had BMI > 40 kg/m^2^, and had cPRA > 50%.

|  |  | Dual | Single | p |
| --- | --- | --- | --- | --- |
| Uncensored Graft Failure | # / N | 28/89 | 570/2201 | - |
|  | Incidence Rate (/100 person-years) | 8.35 | 6.53 | - |
|  | Incidence Rate Ratio | 1.28  (0.87, 1.86) | Reference | 0.21 |
|  | Incidence Rate Ratio (adjusted*) | 0.57  (0.35, 0.93) | Reference | 0.03 |
| Death-censored graft failure | # / N | 12/89 | 278/2201 | - |
|  | Incidence Rate (/100 person-years) | 3.58 | 3.19 | - |
|  | Incidence Rate Ratio | 1.11  (0.62, 1.98) | Reference | 0.72 |
|  | Incidence Rate Ratio (adjusted*) | 0.31  (0.13, 0.71) | Reference | 0.006 |
| Death with a Functioning Graft | # / N | 16/89 | 292/2201 | - |
|  | Incidence Rate (/100 person-years) | 4.77 | 3.35 | - |
|  | Incidence Rate Ratio | 1.43  (0.87, 2.37) | Reference | 0.16 |
|  | Incidence Rate Ratio (adjusted*) | 0.93  (0.50, 1.71) | Reference | 0.80 |
| Acute Rejection | # / N | 8/89 | 436/2201 | - |
|  | Incidence Rate (/100 person-years) | 2.48 | 5.81 | - |
|  | Incidence Rate Ratio | 0.43  (0.22, 0.87) | Reference | 0.02 |
|  | Incidence Rate Ratio (adjusted*) | 0.2  (0.10, 0.62) | Reference | 0.003 |
